# Early drain site tumor recurrence post adjuvant chemotherapy for locally advanced colon carcinoma: Case report and literature review

**DOI:** 10.1016/j.ijscr.2024.110163

**Published:** 2024-08-13

**Authors:** Ahmed Ltaimi, Anis Hasnaoui, Wissem Triki, Oussema Baraket, Sami Bouchoucha

**Affiliations:** a Faculty of Medicine of Tunis, Tunis El Manar University, Rue Djebal Lakhdar, 1006 Tunis, Tunisia; b Department of General Surgery, Habib Bougatfa Hospital, Tunisia; c Department of General Surgery, Menzel Bourguiba Hospital, Tunisia

**Keywords:** Drain site recurrence, Colorectal cancer, Colloid carcinoma, Chemotherapy, Metastasis

## Abstract

**Introduction:**

Colon carcinoma is the most common type of gastro-intestinal cancer. Despite radical surgery, locoregional recurrence has been observed in 4–11.5 % of patients. Abdominal wall metastasis at the drainage site is an extremely rare finding and only a few cases are described in the literature. The mechanism of this metastasis is unknown, and its management remains unclear due to the rarity of the condition.

**Case presentation:**

A 66-year-old patient underwent left colectomy for locally advanced colonic adenocarcinoma. Eight months after the end of adjuvant chemotherapy, the patient complained of a progressive mass of the left lumbar centered on the previous drain site scar. Abdominal wall recurrence was suspected. The patient had R0 mass excision. Histopathologic examination showed a parietal infiltration by a colloid adenocarcinoma. The patient underwent adjuvant chemotherapy. No recurrence was observed.

**Discussion:**

Since 1999 only six cases of colon cancer drainage site metastasis have been reported. Metachronous solitary abdominal wall metastasis after radical colectomy may occur via cancer cell implantation, lymphatic or hematogenous route, or direct invasion. In case of drain site metastasis, the most likely hypothesis is the implantation of tumor cells into the abdominal wall through the drainage route performed during surgery.

**Conclusion:**

The appearance of abdominal wall mass after colon cancer resection must always be considered suspicious. To reduce the risk of abdominal wall metastasis we recommend minimizing tumor manipulation, resection the route of previous percutaneous drainage and performing a radical surgery. Metastasis resection combined with chemotherapy is the appropriate approach to treat these metastases.

## Highlights

• Metachronous solitary abdominal wall metastasis after radical colectomy is a rare finding.
• The appearance of abdominal wall mass after colon cancer resection must always be considered as a recurrence.
• To reduce the risk of abdominal wall metastasis, resect the pathway of prior percutaneous drainage and perform radical surgery.
• Metastasis resection with negative margins plus chemotherapy is the best approach for treating abdominal wall metastasis.

## 1 Introduction

Colon carcinoma is the most common type of gastro-intestinal cancer. Radical surgery is the only curative treatment. The prognosis of colon carcinoma is improved on the one hand, by standardizing the surgical procedure and, on the other hand, by the development of chemotherapy. Despite therapeutic advances in the treatment of colon cancers, the occurrence of locoregional or distant recurrence is observed after radical treatment. Locoregional recurrence has been observed in 4–11.5 % of patients who have undergone curative resection [1]. Advanced disease stage, lympho-vascular invasion, involved resection margins, primary tumor location, and the discovery of the tumor by a complication such as perforation were independent risk factors of recurrence [2]. Metastasis sites are generally the liver, digestive anastomosis, or peritoneum. Abdominal wall metastasis at the drainage site is an extremely rare finding and only a few cases are described in the literature. The mechanism of these metastasis is unknown, and its management remains unclear due to the rarity of the pathology. We reported a case of tumor recurrence at the drain site after colectomy for colon adenocarcinoma. This work has been reported in line with the SCARE criteria [3].

## 2 Case presentation

**Fig. 1:**
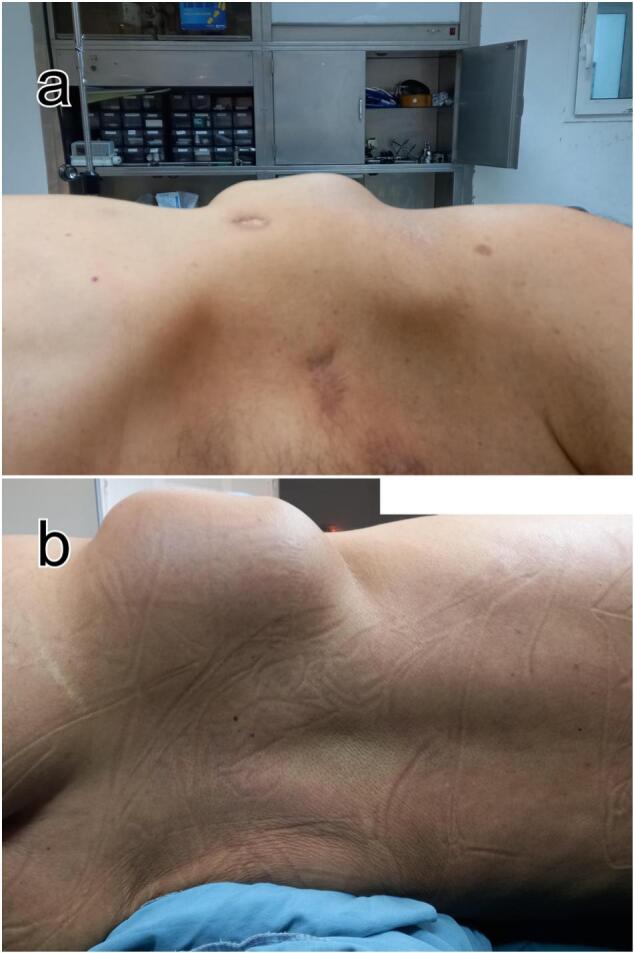
Pre-operative view of the left lumbar mass, a: anterior view, b: posterior view.

**Fig. 2:**
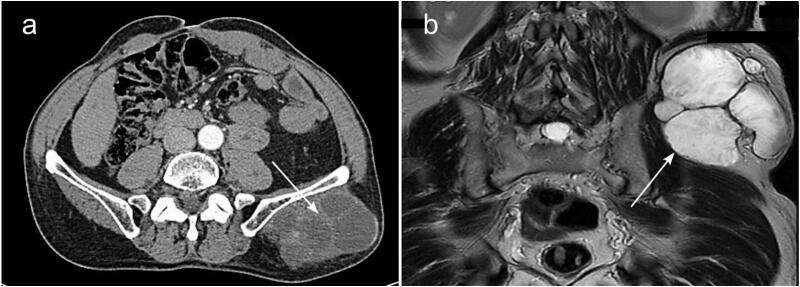
Pre-operative imaging findings. a: Abdominal CT scan with contrast, transverse section passing through the mass (White arrow). b: Coronal view of T2 weighted MRI of the soft tissues showing a cluster of cystic lesions embedded in the subcutaneous fat of the lumbar region (White arrow).

A 66-year-old male patient, with medical history of arterial hypertension, underwent in June 2020 an emergent laparotomy for locally advanced colonic cancer of the splenic flexure. Per operatively, the tumor invaded the abdominal wall and was complicated by a parietal abscess. He had a left colectomy with parietal invasion resection, abscess drainage, and double stoma. Histological examination of the surgical specimen revealed a mucosal colloid adenocarcinoma classified as pT4b N0 (0/25 Nodes) M0, without perineural invasion or vascular emboli. The patient received 4 cycles of adjuvant chemotherapy, specifically FOLFOX (5-fluorouracil and oxaliplatin), which was completed in February 2021 with good tolerance. In June 2021 the patient had a restoration of digestive continuity. A thoraco-abdomino-pelvic CT scan showed no evidence of recurrence, and tumor markers were within normal limits. Eight months after completing chemotherapy (4 months after restoration of digestive continuity), the patient reported a gradually progressive asymptomatic swelling of the left lumbar region (Fig. 1). Upon examination we identified a solid painless mass centered at the previous drain site scar. The mass was well-defined and fixed to the underlying planes. Tumor Markers were elevated, carcinoembryonic antigen: 10.2 ng/ml (0–2.9 ng/ml) and carbohydrate antigen (CA19-9): 43.8 U/ml (<37 U/ml). A thoraco-abdomino-pelvic computerized tomography scan revealed a contiguous cystic formation within the soft tissues of the left gluteal region with exophytic development. This mass measured 12 × 9 × 6 cm, featuring fine calcification along the wall and no enhancement (Fig. 2.a). Magnetic Resonance Imaging of soft tissues showed a cluster of cystic lesions embedded in the subcutaneous fat of the lumbar region and abut the left gluteus maximus muscle without signs of invasion. It measured at 11.5 × 9 × 6 cm, taking the contrast in an annular way after Gadolinium injection, revealing some endoluminal papillary projections, evoking a suspicious nature. These lesions were in clear T1 hyposignal and T2 hypersignal containing linear spans in T2 hyposignal, surrounded by a border of strong T2 hyposignal, T1 FAT SAT hypersignal corresponding to a calcium border (Fig. 2.b).

**Fig. 3:**
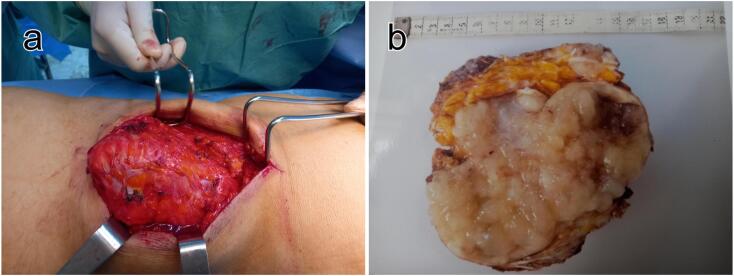
Macroscopic examination after resection. a: Intraoperative view showing the external appearance of the tumor. b: The section of the tumor revealed a yellowish gelatinous content.

The diagnosis of abdominal wall recurrence was suspected. A screening colonoscopy was normal. In particular, there was no anastomotic recurrence. The patient was operated on in June 2022. He was placed in the right lateral decubitus position, and a left lumbar incision was made. Intraoperatively, a lumbar solid and cystic mass invading the gluteal and spinal muscles, and the left iliac crest was found (Fig. 3.a). An R0 resection of the mass was performed, successfully removing the invaded muscle and bone structures. After surgical removal, the closure of the different planes did not require reconstruction. The postoperative course was uneventful. Macroscopic examination of the surgical specimen revealed a yellowish gelatinous tumor neoformation that infiltrates the muscle and parietal fat and measures 13 × 9 cm (Fig. 3.b). Histologically, it consisted of carcinomatous proliferation interspersed with necrosis foci. It was arranged in glandular structures, poly adenoids, spans, cords and isolated cells bathed in an abundant mucoid stroma. It was a parietal infiltration by a moderately differentiated mucosal colloid adenocarcinoma compatible with a colonic origin. The tumor had been completely excised. The patient underwent adjuvant chemotherapy consisting of 6 cycles of FOLFOX. Currently, there is no recurrence at 20 months postoperatively.

## 3 Discussion

This clinical case reports a rare site of metastasis after colectomy for colon cancer and describes our management in the absence of international guidelines. The PubMed, Cochrane, Science Direct and Medline literature databases were searched for literature reviews and case reports published between 1999 and june2024 regarding drain site tumor metastasis after colon carcinoma resection using the following terms: colon cancer, adenocarcinoma, abdominal wall metastasis, drainage site recurrence. Since 1999 only six cases of colon cancer drainage site metastasis have been reported [[4], [5], [6], [7], [8], [9]]. Some cases of abdominal wall metastasis are excluded from this review of the literature because the primary lesion was the rectum or the small intestine. Table 1 presents a summary of the literature review that enables the identification of epidemiological and pathological characteristics associated with this entity.

**Table 1:** Epidemiological, pathological characteristics and management procedure of primary tumor and abdominal wall metastasis.

	Age	Sex	Tumor location	Peroperative findings	Operation	Histopathology	Colon tumor classification	Adjuvant therapy	Time of recurrence (months)	Size of recurrence (cm)	Others metastasis	Treatment of recurrence	Follow-up
Torzilli 1999 [4]	83	F	Ascending colon	NS	Right hemicolectomy	ADK	Dukes'C (stage III)	NS	15	1.6	No	NS	NS
Ilker 2008 [5]	30	M	Ascending colon	Perforated neoplasm	Right hemicolectomy	Mucinous ADK	M0	CT	17	5	Liver metastasis 9 months after colon resection treated by hepatic resection then CT	Surgical removal	NS
Chida 2018 [6]	70	F	Ascending colon	NS	Right hemicolectomy with D3 lymphadenectomy	NS	T4a N1 M0	NS	9	NS	No	Surgical removal	Recurrent tumor in the abdominal wall and anastomotic site 4 months after the second surgery
Polintan 2022 [7]	70	M	Sigmoid	Tumor adherent to urinary bladder	Left hemicolectomy with cystorraphy and omentectomy	Well differentiated ADK	T4b N1 M0 (2N+/11N)	6 cycles of capecitabin and oxaliplatin	12	10 ∗ 8 ∗ 6	No	Surgical removal after CT	NS
Le 2023 [8]	37	M	Transverse colon	Perforated tumor with involvement of stomach	Right hemicolectomy and gastric wedge resection	Moderately differentiated invasive ADK	T4b N0	3 cycles of folfox	<12	3.1 ∗ 1.3	No	Surgical removal	NS
Kowlessar 2024 [9]	50	M	Ileo-caecal valve	Ileo-colic Tumor	Right hemicolectomy and segmental small bowel resection	ADK	NS	CT	>24	NS	Anastomotic recurrence treated by redo ileocolic resection	Surgical removal	No recurrence
Our case 2024	66	M	Splenic flexure	Perforated tumor invaded the abdominal wall and complicated by parietal abscess	Left colectomy with resection of the parietal invasion	Mucosal colloid ADK	T4b N0 M0 (25N-)	4 cycles of folfox	16	13*9*6	No	Surgical removal followed CT	No recurrence

ADK: adenocarcinoma, CT: chemotherapy, F: female, M: male, NS: unspecified.

Both right and left colon are concerned by drain site metastasis, in fact 4 cases of right colon, one case of left colon and one case of transverse colon were documented. All reported cases did not find any metastasis at the time of colon tumor diagnosis and the tumor was classified as M0. However, in all cases the colon tumor was perforated or locally advanced. This finding defends the hypothesis of direct implantation of cancer cells during surgical resection. Thus, it seems that the advanced stage of colon carcinoma is a risk factor for drain site recurrence. All patients received curative resection. Despite adjuvant chemotherapy administered to at least five patients, parietal metastasis was observed. Histological exam of the colon neoplasm revealed an adenocarcinoma in all cases. The delay in the parietal recurrence was between 9 and 24 months after colon resection. Regarding the size of the abdominal wall recurrences, it varies between 2 and 10 cm, our case reports the largest size of recurrence (13 ∗ 9 ∗ 6 cm). The diagnosis of parietal metastasis led to the carrying out of a complete extension workup that did not identify other sites of recurrence, particularly at the level of the anastomosis site. In two cases [5,9] a liver metastasis and anastomotic site recurrence were documented before wall recurrence and were treated respectively by liver resection and redo ileocolic resection. All cases of parietal metastasis were treated by surgical removal. In another case [7] the resection was preceded by chemotherapy (irinotecan and capecitabine). In the absence of guidelines, we choose to administer adjuvant chemotherapy in our case. After surgical removal of parietal recurrence, a case of second recurrence [6] was reported four months later, the tumor recurred at the same site of abdominal wall and at the anastomotic site. The occurrence of a parietal recurrence at the drainage site is an extremely rare entity, unlike other locoregional recurrences, the mechanism of occurrence of this type of recurrence remains poorly understood. Metachronous solitary abdominal wall metastasis after radical colectomy may occur via cancer cell implantation, lymphatic or hematogenous route, or direct invasion [10]. In case of drain site metastasis, the most likely hypothesis is the implantation of tumor cells into the abdominal wall through the drainage route performed during surgery. Therefore, we recommend minimizing tumor manipulation during colectomy, resecting the pathway of previous percutaneous drainage and performing radical surgery in cases of locally advanced tumors, leaving no residual disease.

Clinical presentation of drain site metastasis is often an abdominal mass at the drainage site. This mass can be painful and gradually increases in size. Tumor Markers increasing is very suggestive of recurrence. Percutaneous biopsy of the mass can confirm the diagnosis of malignancy, but a negative biopsy does not rule out recurrence. Therefore, it is important to consider any mass that appears at the drainage site as a potential recurrence and to carry out the necessary investigations to confirm or rule out the diagnosis. Abdominal wall metastasis are often indicators of recurrent intra-abdominal cancer. Patients who present this type of recurrence require a complete staging workup that includes physical examination, total colonoscopy, measurement of tumor markers, and a computerized tomography scan.

However, due to the very low incidence of abdominal wall recurrence, there is no specific management recommendation currently available. Metastasis resection with negative margins combined with chemotherapy is the most appropriate approach. Radiotherapy seems an interesting alternative, but it has not been evaluated in this context. Some authors suggest that radiotherapy is not recommended when a complete excision has been achieved [11].

Our study has some limitations. In fact, the rarity of this type of recurrence limits the generalizability of findings, and conclusions drawn from a single case may not apply universally to all similar cases.

## 4 Conclusion

The appearance of abdominal wall mass after colon cancer resection must always be considered suspicious, especially if the tumor was locally advanced. The most likely hypothesis is the implantation of tumor cells into the abdominal wall during colectomy. To reduce the risk of abdominal wall metastasis we recommend minimizing tumor manipulation during colectomy, resecting the pathway of a previous percutaneous drainage and performing a radical surgery in cases of locally advanced tumors. Metastasis resection with negative margins combined with chemotherapy is the most appropriate approach to treat this condition.

## Consent

Written informed consent was obtained from the patient for publication and any accompanying images. A copy of the written consent is available for review by the Editor-in-Chief of this journal on request.

## Ethical approval

Ethical approval was deemed unnecessary by the institutional ethics committee of Bougatfa Hospital, Bizerta, Tunisia, as the paper is reporting a single case that emerged during normal practice. Ethical approval is not required in our institution for case reports.

## Funding

This research did not receive any specific grant from funding agencies in the public, commercial, or not-for-profit sectors.

## Author contribution

Ahmed ltaimi: Conceptualization, writing-Original draft preparation. Anis Hasnaoui: Writing-Reviewing and Editing. Wissem Triki: Data curation. Oussema Baraket: Writing-Reviewing. Sami Bouchoucha: Writing-Reviewing. All authors read and approved the final manuscript.

## Guarantor

Anis Hasnaoui.

## Conflict of interest statement

Nothing to declare.
